# Getting to grips with ammonium

**DOI:** 10.7554/eLife.01029

**Published:** 2013-07-02

**Authors:** Yi Wang, Jonathan A Javitch

**Affiliations:** 1 **Yi Wang** is in the Department of Psychiatry and the Center for Molecular Recognition, Columbia University, New York, United States, and the Division of Molecular Therapeutics, New York State Psychiatric Institute, New York, United Statesyw2483@columbia.edu; 2 **Jonathan A Javitch** is in the Departments of Psychiatry and Pharmacology and the Center for Molecular Recognition, Columbia University, New York, United States, and the Division of Molecular Therapeutics, New York State Psychiatric Institute, New York, United Statesjavitch@nyspi.columbia.edu

**Keywords:** transport, transport proteins, biosensor, ammonium, GFP, fluorescent probes, Arabidopsis

## Abstract

A fluorescent sensor that can monitor levels of extracellular ammonium has been made by using a fused green fluorescent protein to detect conformational changes in ammonium transport proteins.

**Related research article** De Michele R, Ast C, Loqué D, Ho C-H, Andrade SLA, Lauquar V, Grossmann G, Gehne S, Kumke MU, Frommer WB. 2013. Fluorescent sensors reporting the activity of ammonium transceptors in live cells. *eLife* **2**:e00800. doi: 10.7554/eLife.00800

**Image** A biosensor for extracellular ammonium made by inserting a modified green fluorescent protein (bottom) into an ammonium transport protein (top)

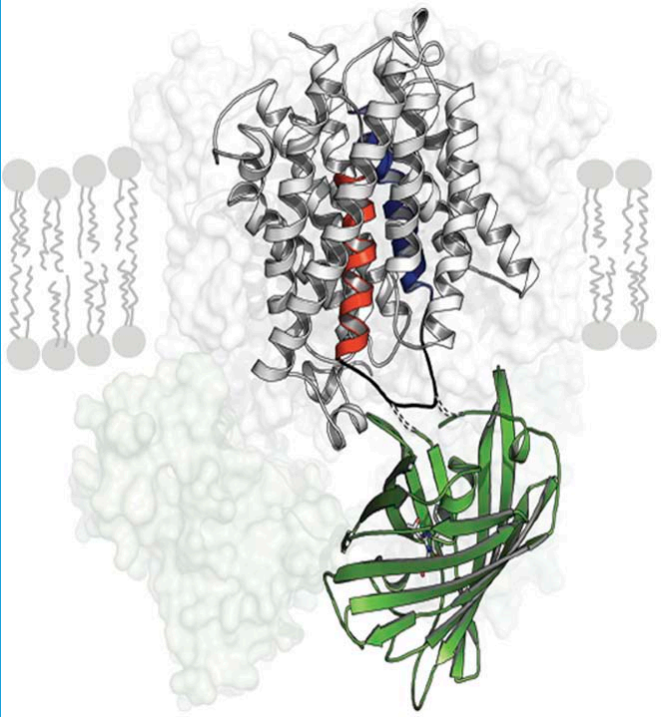

All organisms require nitrogen for their survival. However, high levels of nitrogen can be toxic to cells, so its uptake must be tightly controlled, although the mechanisms responsible for controlling nitrogen levels in cells are poorly understood (von Wirén and Merrick, 2004). In nature, nitrogen is frequently found in inaccessible forms that require conversion before use by the cell. Ammonia (NH_3_) and ammonium (NH_4_^+^) are major sources of nitrogen in bacteria, fungi and plants; both are readily metabolized, but the latter is much more prevalent at cellular pH.

Ammonium can enter cells with the help of specific transport proteins or via a number of channels through which other ions and/or water molecules can also pass. However, existing experimental techniques have not been able to determine the relative contributions made by transport proteins and channels to the uptake of ammonium by plant cells. Moreover, these techniques have lacked the spatial and temporal resolution to study the workings of ammonium transport proteins in living cells. Now, in *eLife*, Wolf Frommer of the Carnegie Institution for Science and co-workers—including Roberto De Michele as first author—have developed a fluorescent biosensor to study ammonium transport proteins in vivo using fluorescence microscopy (De Michele et al., 2013).

The ammonium transport proteins found in plants belong to a superfamily of such proteins that is conserved in bacteria, plants and animals. In general these proteins contain 11 or 12 transmembrane helices, which can be divided into two halves. As is the case with many transport proteins, these halves are structurally similar but organized with pseudo-twofold symmetry as an inverted repeat (Forrest, 2013; Shi, 2013; Figure 1). High-resolution atomic structures have been obtained for two ammonium transport proteins—AmtB, which is found in bacteria (Khademi et al., 2004), and AMT-1, which is found in Archaea (Andrade et al., 2005)—and these structures indicate that the ammonium is transported along a pathway that lies between the two halves of the protein. Several residues situated in the fifth and sixth helices, and the highly mobile cytosolic loop that connects these two helices, are predicted to be involved in the recognition and transport of the ammonium (Andrade et al., 2005), but the details of the transport mechanism and its regulation are not fully understood.

**Figure 1.**
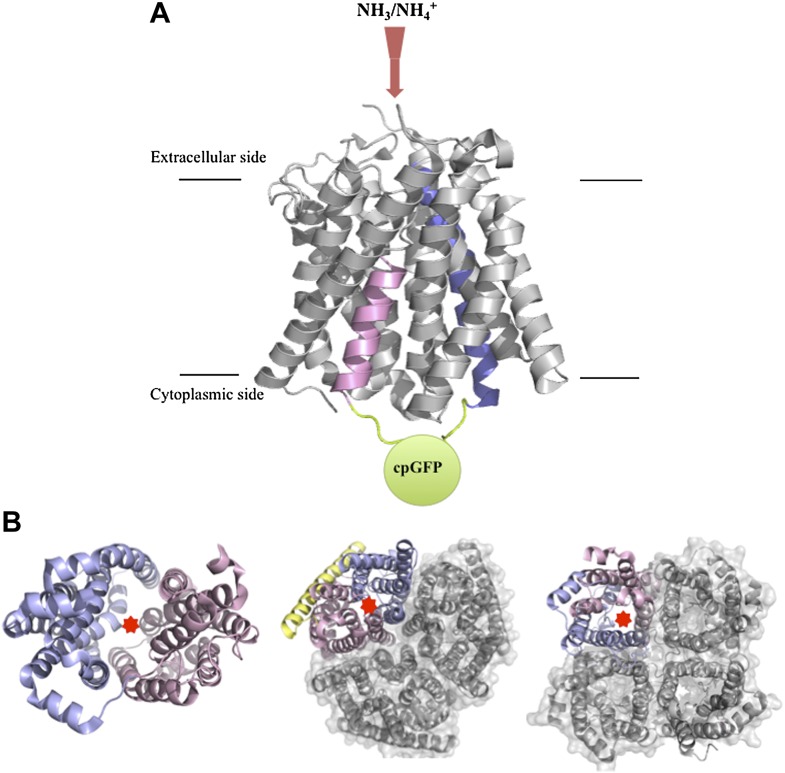
Structures of transport and channel proteins. (**A**) Representation of the ammonium transport protein studied by Frommer and co-workers (De Michele et al., 2013). This protein, which contains 11 transmembrane helices, transports ammonium into the cell (red arrow). De Michele et al. inserted a variant of green fluorescent protein (cpGFP; green) into the cytosolic loop between helix V (shown in pink) and helix VI (blue) to create a fluorescent sensor that reports the binding of ammonium to the protein and/or the transport of ammonium through the internal pore. (**B**) Representative transport and channel proteins that share an internal pseudo-twofold symmetry. LacY (left; Abramson et al., 2003) and AmtB (centre; Khademi et al., 2004) are transport proteins; human aquaporin 5 (right; Horsefield et al., 2008) is a channel protein. All three are shown as ribbon diagrams viewed from the cytoplasmic side: light pink indicates the N-terminal half and light blue indicates the C-terminal half; the yellow ribbon in AmtB is not part of either half. In each protein, the substrate (lactose, ammonium or water) is thought to be translocated through the interface between the two halves (marked by red stars). AmtB and AQP5 can assemble in the plasma membrane into compact trimers and tetramers respectively (grey). Figure prepared by Yi Wang based on images from the Protein Data Bank: www.rcsb.org/pdb/home/home.do.

Frommer and co-workers—who are based at labs in Germany, Italy and the US—built a fluorescent sensor for ammonium binding/transport by introducing modified green fluorescent protein (GFP) into AMT1;3, an ammonium transport protein found in the plant model organism *Arabidopsis*. GFP and its derivatives generate fluorescence when they are excited by light of the appropriate wavelength, so if they are fused to a protein, they can act as markers, with the fluorescence indicating the location of the protein. The part of the GFP that emits the fluorescence is called a chromophore.

De Michele et al. used a variant of GFP in which the protein sequence is rearranged to make the chromophore more sensitive to the conformation of the protein to which it is attached. When this variant, which is called ‘circularly permutated’ GFP (cpGFP), is fused to a protein at a suitable site, the chromophore can report on conformational changes by increasing or decreasing the level of its fluorescence (Baird et al., 1999; Nagai et al., 2001). They also created two other ammonium sensors: one was based on another transport protein found in *Arabidopsis*, and the other was based on an ammonium transport protein called MEP that is found in yeast. The fact that other sensors could be produced using the same strategy suggests that this approach may be generally applicable.

To develop a biosensor, De Michele et al. introduced cpGFP into several cytosolic regions of AMT1;3. To assess ammonium transport ability, they knocked out the endogenous MEPs in a strain of yeast and then introduced the AMT1;3-cpGFP fusions. Ammonium was still translocated when cpGFP was inserted into the cytosolic loop between the fifth and sixth helices (see Figure 1), but not when it was inserted into other regions. However, the addition of ammonium did not alter the fluorescence. The researchers carried out intensive optimization of the linker sequences just preceding and following the cpGFP, and this enabled them to finally produce a sensor that was able to report changes in the levels of extracellular ammonium in living cells via substantial changes in the intensity of its fluorescence.

De Michele et al. expressed this construct, which they call AmTrac, in oocytes from *Xenopus* (a type of frog) in order to test its ability to report ammonium binding and translocation. They found that the changes in the fluorescence intensity resulting from different levels of extracellular ammonium, as measured by the AmTrac sensor, correlated with levels of ammonium transport determined from measurements of substrate currents. In addition, the affinity constant of AmTrac for NH_4_Cl determined in this way is almost the same as that measured in *Arabidopsis* roots expressing wildtype AMT1;3 (Yuan et al., 2007), suggesting that AmTrac maintains its functional properties. Furthermore, the fluorescence intensity responses of AmTrac showed the expected preference for NH_4_^+^ over other cations.

De Michele et al. moved back to yeast to confirm the strict link between ammonium binding/transport and the level of fluorescence. They reasoned that AmTrac could be reporting ammonium uptake in two ways. First, AmTrac might respond to extracellular ammonium binding or transport that occurs during normal ammonium translocation: this response would involve a conformational change that leads to a change in the level of fluorescence. Alternatively, fluorescence might result from sensing increased cytosolic ammonium not necessarily imported through AmTrac itself.

To test these possibilities, they introduced single-point mutations into AmTrac to inactivate its transport properties without disrupting its localization at the cell membrane, and then expressed these mutant forms in wildtype yeast and also in a strain lacking endogenous MEPs. No changes in the fluorescence intensity were seen in either strain, which rules out the possibility that cytosolic ammonium imported by endogenous MEPs or other transport proteins was responsible for the changes in fluorescence seen in the other experiments. They then introduced further mutations in order to identify a ‘gain of function’ mutation that could restore the ability to bind and transport ammonium, and found that the ammonium-induced changes in the fluorescence intensity were also restored by these mutations. Together these results suggest that fluorescence intensity changes in AmTrac specifically correspond with a conformational change that is associated with the binding or transport of extracellular ammonium.

A remaining challenge is to discriminate between the changes in fluorescence intensity triggered by the binding of ammonium and those triggered by its transport, and thus to understand just what is sensed by this biosensor. The fluorescence might be independently or jointly influenced by a variety of factors: ammonium binding; discrete steps (or subsets of steps) during transport; or the full transport cycle. Still, for many years the technology to study plant transport proteins has been restricted to electrophysiological methods and in vitro uptake assays that cannot capture their activity or regulation in living cells. Now, empowered by these novel fluorescent activity sensors, it will be possible to study how these transport proteins function in vivo.
